# Comparing traditional and participatory dissemination of a shared decision making intervention (ADAPT-NC): a cluster randomized trial

**DOI:** 10.1186/s13012-014-0158-0

**Published:** 2014-10-29

**Authors:** Hazel Tapp, Andrew McWilliams, Thomas Ludden, Lindsay Kuhn, Yhenneko Taylor, Thamara Alkhazraji, Jacquie Halladay, Diane Derkowski, Sveta Mohanan, Michael Dulin

**Affiliations:** Department of Family Medicine, Carolinas HealthCare System, 2001 Vail Avenue, Suite 400 Mercy Medical Plaza, Charlotte, NC 28207 USA; Dickson Advanced Analytics, Carolinas HealthCare System, Charlotte, USA; Department of Family Medicine, UNC Chapel Hill and The Cecil G. Sheps Center for Health Services Research, Chapel Hill, USA; Carolinas Medical Center, Charlotte, USA

**Keywords:** Asthma, Shared decision making, Facilitated implementation, Dissemination, Decision aids

## Abstract

**Background:**

Asthma is a common disease that affects people of all ages and has significant morbidity and mortality. Poor outcomes and health disparities related to asthma result in part from the difficulty of disseminating new evidence and care delivery methods such as shared decision making (SDM) into clinical practice.

This 3-year study explores the ideal framework for rapid dissemination of an evidence-based SDM toolkit for asthma management. The study leverages a partnership between the North Carolina (NC) statewide Medicaid network and the NC Network Consortium of practice-based research networks (PBRNs).

**Methods/design:**

This non-blinded study will randomize 30 primary care clinics in NC stratified by four PBRNs. We will test dissemination across these practices using a facilitator-led participatory approach to dissemination (FLOW), a novel method of participatory dissemination involving key principles of community-based participatory research, and a more typical “lunch and learn” dissemination method. Specifically, we will use cluster randomization to assign each of the 30 practices to one of three arms: (1) control, no dissemination; (2) traditional dissemination, one didactic session a year and distribution of educational material; and (3) FLOW dissemination. We hypothesize that at the unit of randomization, the clinic, patients in the FLOW dissemination arm will be more likely to share in their treatment decisions compared to patients in the traditional dissemination or control arms. All outcomes will be measured at the level of the clinic. Adoption of the SDM approach will be evaluated by 1) asthma exacerbations, 2) level of patient involvement in the decision making process, and 3) qualitative assessments from patients and providers.

The research question is: What dissemination strategy most effectively increases practice level adoption of a shared decision making approach to asthma management? This study will provide important data to support best practices in dissemination of an evidence-based toolkit and implementation of shared decision making into primary care practices.

**Trial registration:**

The trial was registered on January 27, 2014 through the United States National Institutes of Health’s ClinicalTrials.gov NCT02047929 and funded by the Patient-Centered Outcomes Research Institute (PCORI).

## Background

Asthma is an inflammatory lung disease that affects people of all ages and has significant morbidity and mortality. In the US, asthma affects over 26 million people and has experienced a concerning increase in overall prevalence [1,2]. Despite evidence that this disease can be managed on an outpatient basis, the burden of asthma remains high, and this condition alone is responsible for 2 million emergency department (ED) visits, 439,000 hospitalizations, and 3,000 deaths every year [3]. In addition to its effect on increased health-care utilization, asthma negatively impacts patients’ quality of life. Over 20% of all asthma patients miss at least 1 day of work or school every year, and twice as many rate their health as poor compared with the general population [4]. There are also marked disparities in asthma outcomes for vulnerable populations. For example, African-American children with asthma have three times the rates of their Caucasian counterparts in hospitalizations and ED utilization, and their mortality rates are almost five times higher [5].

Poor outcomes and disparities for patients with asthma persist despite advances in medical knowledge. For example, the use of self-management tools and shared decision making (SDM) has produced notable positive changes in asthma outcomes; however, uptake of these new approaches has been slow [6,7]. Widespread adoption is lacking in part because of the gap in understanding how best to disseminate evidence-based interventions into everyday practice. Indeed, dissemination of information and interventions into practice has been highlighted as a key national priority by the Patient-Centered Outcomes Research Institute (PCORI), the Agency for Healthcare Research and Quality (AHRQ) and the Institute of Medicine (IOM) [8,9]. Despite this focus, the field of dissemination research is relatively new. Known barriers to dissemination include heterogeneous patient and provider populations, limited support staff, lack of clinic resources, pressure on practices to improve efficiency, and the complexity of electronic medical record (EMR) systems [10-12]. The most common type of dissemination is the traditional model (active diffusion), which does not adequately overcome these barriers. This process includes exposure to academic detailing by subject matter experts, journal publications, didactic presentations, and educational material distributed in paper and online formats [13,14].

Shared decision making represents a complex intervention that exemplifies the significant impact of barriers to adoption. Key barriers to implementation and sustainability include practice flexibility, provider and staff beliefs, attitudes, and motivations [15,16]. Providers typically perceive barriers to adoption, including time constraints and concern that SDM may not be applicable to their practice’s patient population. These perceptions may be because of the patients’ limited education or a preference that all medical decisions should be made by their physician [15,17,18]. Despite these concerns, providers tend to feel that incorporating SDM into their practices will improve patient outcomes and satisfaction with their care [17,18].

To address potential problems with the spread of new evidence-based practices like SDM, we previously studied implementation of asthma SDM across six primary care sites and identified best practices for studying dissemination [19,20]. Using key principles of community-based participatory research (CBPR) to engage practice stakeholders and patients, we developed a facilitator-led approach to dissemination, called facilitator-led participant owned (FLOW) dissemination. Also, as part of this dissemination pilot, we established dissemination evaluation methods using quantitative outcomes data (ED visits, hospitalizations, and prescriptions for oral steroids), as well as qualitative evaluation of provider and patient satisfaction. As a next step, we took the lessons learned from this smaller scale dissemination pilot and adapted them to this larger comparative effectiveness study of dissemination methods.

The ideal setting for a pragmatic study such as this is within practice-based research networks (PBRNs) [21,22]. The current study will occur within a well-established consortium of four PBRNs called the North Carolina Network Consortium (NCNC). NCNC is a “meta” network of investigators, community members, and primary care practices affiliated with Duke University, the University of North Carolina (UNC) at Chapel Hill, East Carolina University/Vidant Health and Carolinas HealthCare System.

### Asthma SDM toolkit

The previously described Asthma SDM Toolkit that will be used in the current study is based upon the Better Outcomes for Asthma Treatment (BOAT) study [7]. The SDM Toolkit used in this project includes (1) a tool to assess baseline asthma control, (2) a guide for eliciting the patient’s goals and priorities around medication options, (3) asthma educational materials, and (4) a tool to guide the negotiation process to jointly develop a treatment regimen that accommodates the patient’s goals and preferences. At the conclusion, an asthma action plan is provided. The SDM Toolkit, implementation resources, and a training video can be found at: https://asthma.carolinashealthcare.org/.

### Study objectives

The specific objectives of this study to be examined at the clinic level are:

1. To compare the effectiveness of traditional and participatory dissemination models for a shared decision making intervention.
2. To determine which dissemination strategy results in practices most effectively adopting a shared decision making approach to asthma management.

## Methods/design

This study received ethics approval from the Institutional Review Board of Carolinas HealthCare System.

### Description of interventions

**Figure 1:**
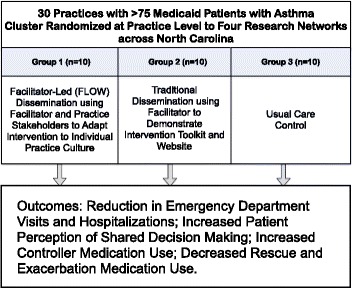
**Study design.**

**Figure 2:**
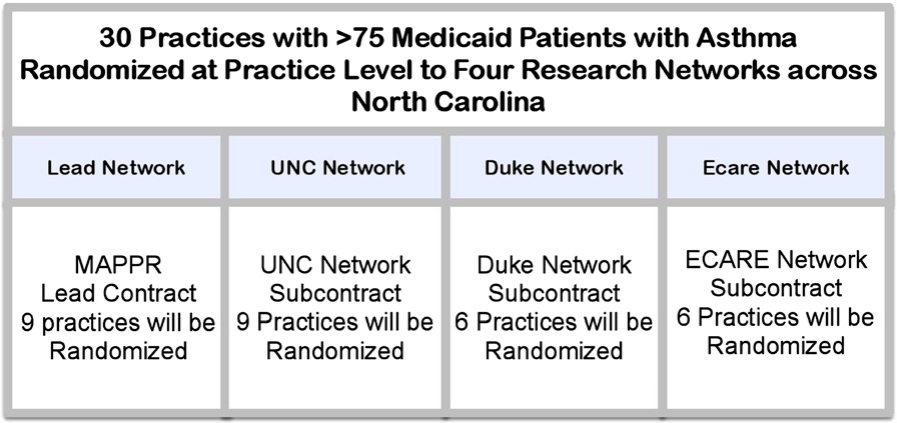
**Practice based networks and cluster randomization.**

This study compares ways to disseminate evidence-based interventions to practices using practice-level dissemination methods. Each dissemination method is designed for practice-wide adoption, and as such, the study will use a practice-level cluster randomized controlled design. The clinics will be stratified based on geographical location within North Carolina. To meet the inclusion criteria, all clinics will have at least 75 patients aged 5–40 with Medicaid coverage and asthma defined by one or more of the following criteria: 1) inpatient visit with an asthma primary diagnosis, 2) emergency department visit with an asthma primary diagnosis, 3) at least four outpatient visits with asthma primary diagnosis and at least two asthma medications, or 4) four asthma medications. Patients with a diagnosis of chronic obstructive pulmonary disease (COPD) will be excluded from the quantitative analyses. All asthma patients at the clinics will be eligible for the intervention, while only the Medicaid patients meeting the above criteria will be included in the exacerbation analysis. Thirty diverse primary care clinics will be recruited from a state-wide pool of eligible clinics geographically accessible to each of the four PBRNs (Figure 1). The first 30 consenting practices will be equally randomized into one of three study arms: (1) facilitator-led participant owned (FLOW) dissemination, (2) traditional dissemination, and (3) control. The cluster randomization of clinics by participating PBRNs (Figure 2) will ensure relatively equal geographical distribution.

### Facilitator-led approach to dissemination

For this study arm, the facilitator joins the practice for approximately 1 hour each week over a 12-week period to train all staff on different components of the SDM process. Training includes introduction to the FLOW approach, an overview of the Asthma SDM Toolkit, scheduling logistics for each practice, patient recruitment strategies, toolkit training, and role play of the patient-provider interactions. Training also utilizes videos showing providers using the SDM techniques. Generally, the first patients are expected to be seen around week 8 of the process. Remaining visits cover feedback from patient visits and troubleshooting. FLOW arm clinics will be invited to join a monthly conference call with the other FLOW clinics to share best practices and discuss logistics.

### Traditional dissemination

Practices randomized to traditional dissemination receive two lunchtime presentations on shared decision making separated by 12 months. The facilitator gives an overview of the Asthma SDM Toolkit, access to the website with additional information, and a copy of all printed materials associated with the SDM Toolkit.

### Control

A third group will be randomized into an arm with no formal dissemination. This arm will receive information only through passive exposure to the concepts of shared decision making during practice recruitment, publications, conferences, or word of mouth. At the conclusion of the project, practices in the control arm will be introduced to the same study materials given to the FLOW and traditional arms.

### Focus groups

The research team and practice facilitators will conduct focus groups consisting of providers, staff, and patients from practices in each of the three arms. The 8–10 participants in each focus group will be asked about SDM within their practice. The focus groups will solicit feedback from the stakeholders regarding their perceptions of the dissemination process and the Asthma SDM Toolkit. Providers will be asked about their perceptions of the study and its impact on their ability to provide high-quality asthma care. In addition, patients will provide qualitative assessments of their perceived involvement in the asthma care decision making process.

A total of six focus groups will be conducted at each PBRN involving all three arms of the study. Four focus groups will be conducted with participants in the FLOW arm with one focus group occurring every 6 months of the study. The traditional arm will conduct one focus group within 12 months of the intervention delivery and concurrent with one of the 6-month intervals. Lastly, the control arm will have one focus group starting in year 2 of the study.

### Survey

Clinics in arms 1 (FLOW) and 2 (traditional) of the study will be asked to collect the ongoing surveys to determine their patients’ level of involvement in asthma care decisions. The survey consists of one question asking “who made the decision about what your asthma treatment would be? Was it you alone, the provider alone, or partly or equally shared between provider and patient?”.

### Setting

**Figure 3:**
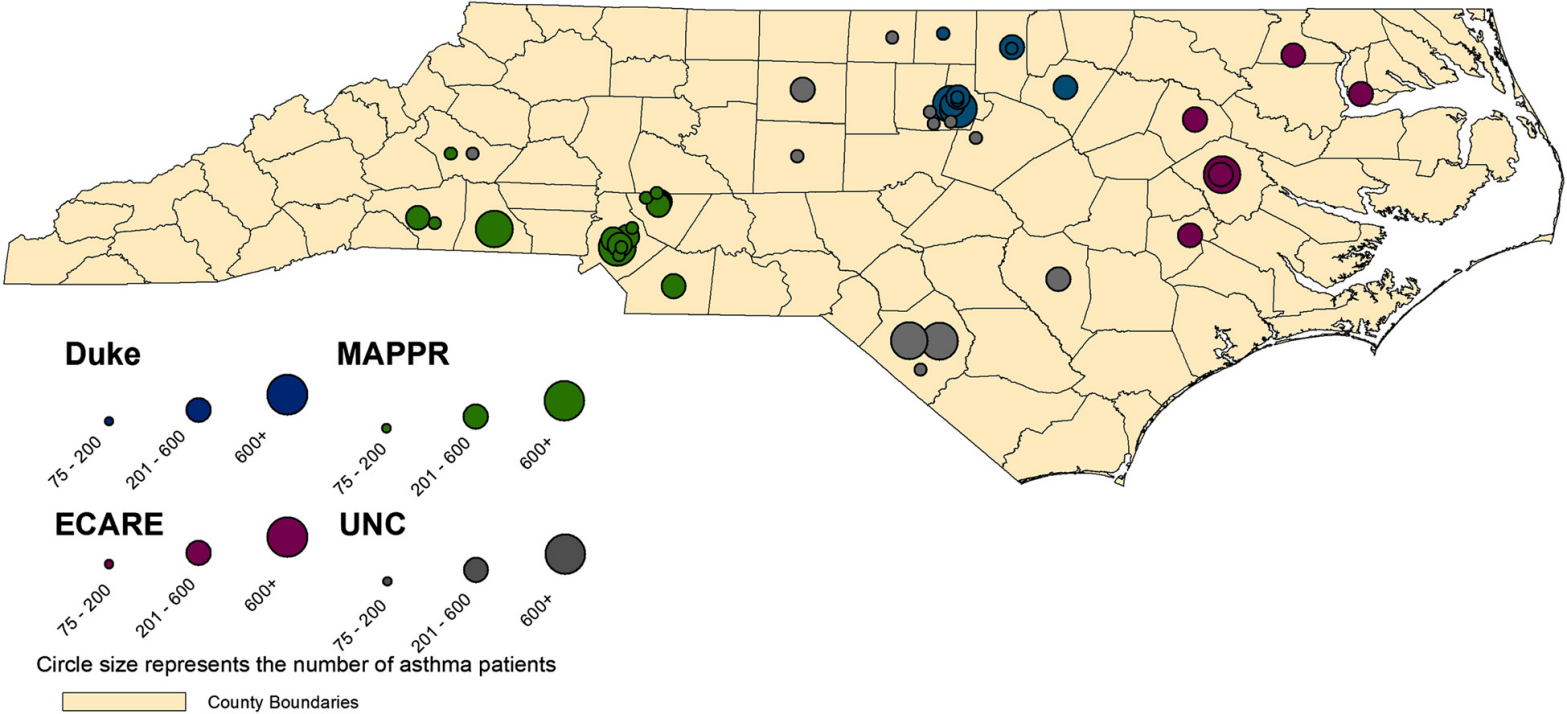
**MAP of practices meeting study criteria.**

The NCNC network includes diverse practices that range in size, location, practice type, and the race/ethnicity of their patients (Figure 3 and Table 1).

**Table 1:** **Practice-based research networks in the North Carolina Network Consortium**

**Network name**	**Total number of practices**	**Brief description, types of practices, and population**
Mecklenburg Area Partnership for Primary Care Research (MAPPR)	85	Lead for proposal
Lead contract		Practices affiliated with a large hospital system, (CHS) Pediatrics, Internal Medicine, Family Medicine
		Population: urban and rural; all ages; African-American and Latino
UNC affiliated networks:	114	Statewide primary care network
North Carolina Family Medicine Research (NC-FM-RN)		Pediatrics, Internal Medicine, Family Medicine
North Carolina Multisite Adolescent Research Coalition for Health (NC-MARCH)		Population: urban and rural; all ages; Native American, African-American, and Latino
Subcontract		
Duke Primary Care (PCRC)	34	Duke Health affiliated practices
Subcontract		Internal Medicine, Family Medicine, Pediatrics
		Population: all ages
Eastern Carolina Association for Research and Education (E-CARE)	6	Practices affiliated with a large hospital-system
Subcontract		Pediatrics, Internal Medicine, Family Medicine
		Population: rural health; all ages; largely African-American

Descriptions of patient population and types of practices within each network.

The primary mission of NCNC is to address pressing questions related to the delivery of primary care health services and the management of primary care problems. NCNC represents over 300 practices with over 1,400 health-care providers in 52 counties across the state and includes family medicine, internal medicine, and pediatric clinics. The practices within NCNC 1) range in size from those run by solo practitioners to large community health centers; 2) are located in urban, rural, and suburban areas; and 3) have patients who reflect the diversity of the state’s minority and underserved populations including Latinos, African-Americans, Native Americans, and other vulnerable populations. NCNC engages primary care practices in quality improvement and research spanning from observational studies to clinical trials but also provides direct practice support via creating and conducting on site continuing medical education and training curricula for practice staff and providers [19].

In 2011, Medicaid covered approximately 1.5 million non-elderly individuals in North Carolina with a racial breakdown of 43% White, 35% Black, and 14% Hispanic [23]. Practices accepting Medicaid patients are located in each of North Carolina’s 100 counties, and thus, the population is geographically heterogeneous with both rural and urban representations.

The 30 practices will be recruited by the four PBRNs either from practices within their existing network or practices within an appropriate geographically defined area. Each PBRN has identified practices that meet the study’s inclusion criteria. Individual PBRNs will be responsible for recruiting two or three practices per arm for a total of six or nine practices per PBRN and will be responsible for recruitment and oversight of a practice facilitator. As the lead group, Mecklenburg Area Partnership for Primary Care Research (MAPPR) will be responsible for hosting a centralized training day and ongoing continuing education for all practice facilitators.

### Analysis

We hypothesize that at the unit of randomization, the clinic, patients in the FLOW approach to dissemination will be more likely to share in their treatment decision compared to patients in the traditional dissemination or usual care arms. Qualitative data will be collected to assist in understanding barriers to implementation, to ensure that patients feel that they are partners in the development of the asthma care plan, and to improve the facilitator-led implementation approach over time.

Also, we hypothesize that clinical outcomes in arm 1 will show greater improvement than arms 2 (traditional) or 3 (control). Bivariate comparisons between study arms will be conducted using chi-square test for proportions and *t*-tests (for two group comparisons) or analysis of variance for means, for each of the six outcome measures: patient perception of shared decision making, ED visits, hospitalizations, controller medication use, beta agonist overuse, and use of oral steroids. Changes over time will be assessed using regression models, including the outcome measure as the dependent variable and study arm, time, and a study arm by time interaction term as independent variables to assess differences by intervention and whether changes over time vary by intervention. Analysis over time will employ generalized estimating equations to address correlation between measures at the same practice [24]. Analysis will be conducted using Statistical Analysis System (SAS) (version 9.3).

Principle investigators at each PBRN site will recruit and enroll clinics into the study. As well as explaining the study and randomization process, each site will provide potential practices with information on incentives for providers and practices such as continuing medical education (CME) category 1 credits for participation and up to 20 CME hours for satisfying MOC4 Metric Module for family medicine providers.

For the purposes of the study, individual participants will be included in a cluster (clinic) by complete enumeration of patients with a diagnosis of asthma. Representatives from each clinic will agree to participate in the study prior to randomization. To ensure that randomization will be non-biased, randomization will occur centrally using a computer-generated algorithm run by the study statistician, who will not be involved in recruitment, data collection, or deployment of the intervention. Clinics will be randomized in groups by PBRN. Once each PBRN reaches its recruitment target, the list of clinics will be inputted into SAS and assignments to one of the three study arms will be generated randomly without replacement using the PROC PLAN procedure. Thereby, allocation to intervention or control group will be concealed from clinics and site PIs prior to full recruitment. Due to the nature of the intervention, which requires active clinic engagement, intervention assignment will be unblinded following randomization.

Sample size was determined using the method of Hayes and Bennett [25] for comparing proportions in clustered randomized trials. We computed the sample necessary to achieve minimum 80% power to test our primary hypothesis that the participatory dissemination strategy will experience greater improvements in patient outcomes compared to traditional dissemination and usual care. A sample size of 10 clinics, with a minimum of 50 patients per clinic, was determined based on preliminary data showing a 7-point decline in the proportion of patients with asthma exacerbation (i.e., ED visit or hospitalization) from 16% to 9% in clinics where the SDM Toolkit was implemented, and a coefficient of variation (*k*) equal to 0.06. To ensure adequate representation of asthma patients during the study period, we approached clinics with at least 75 asthma patients at baseline.

The outcome measure of asthma exacerbation resulting in an emergency department visit or inpatient admission will be determined using Medicaid claims data with the primary diagnosis of asthma (ICD-9 code 493) or a prescription for oral steroids. Misclassification may occur from coding errors but should be non-differential and not vary between the study’s arms since a single source of data will be used for all outcomes (Table 2). National surveillance studies [2] and intervention studies [7,26] have applied asthma ICD-9 codes to administrative data with useful interpretation of outcomes.

**Table 2:** **Measures for assessment of changes in patient asthma outcomes**

**Outcome**	**Data source**	**Study arms and networks**	**Collection frequency**
Patient perception of shared decision making [7]	Survey question “Who made the decision today?” collected via index cards at arm 1 and 2 practices	Arms 1, 2	Bimonthly
Asthma emergency department visits [27]	Medicaid claims, CHS Asthma Comparative Effectiveness Research Database	Arms 1, 2, 3	Quarterly
Asthma hospitalization rate [27]	Medicaid claims, CHS Asthma Comparative Effectiveness Research Database	Arms 1, 2, 3	Quarterly
Controller medication use	Medicaid claims	Arms 1, 2, 3	Quarterly
Beta agonist overuse [28]	Medicaid claims	Arms 1, 2, 3	Quarterly
Exacerbation requiring oral steroid [27]	Medicaid claims, CHS Asthma Comparative Effectiveness Research Database	Arms 1, 2, 3	Quarterly

Data was obtained from surveys, insurance claims, and research databases.

## Discussion

Variation in primary care clinics and providers makes it challenging to implement new practices and concepts around asthma management such as SDM. Identifying facilitation methods that improve the dissemination of interventions is an important step towards advancing patient outcomes and avoiding preventable ED visits and hospitalizations, while simultaneously reducing overall health-care costs. This study is designed to explore and scrutinize the effectiveness of two different implementation methods in a cluster randomized trial with a control arm. Data collected during the study and assessment of the impact of the dissemination strategies will allow the team to develop a theoretical framework to better describe the underlying mechanism supporting clinic practice change.

The innovative FLOW method of dissemination uses a flexible, participatory approach to implementation [20]. This model has demonstrated adaptability to the varied work environments and patient populations of different practices. Inclusion of the traditional dissemination and passive dissemination control arms provides methodological rigor.

The results of this study will aid in identifying the most effective method of dissemination by testing pragmatic interventions in a real world, practice-based setting. This study has the potential to make significant impacts on asthma management through the use of a shared decision making approach to treatment, and, by leveraging a partnership with the state-wide Medicaid network of practices and the consortium of research networks.

## Abbreviations

AHRQ: Agency for Healthcare Research and Quality
BOAT: Better Outcomes for Asthma Treatment
CBPR: community-based participatory research
ED: emergency department
EMR: electronic medical record
FLOW: facilitator-led participant owned dissemination
IOM: Institute of Medicine
MAPPR: Mecklenburg Area Partnership for Primary Care Research
NCNC: North Carolina Network Consortium
PBRNs: practice-based research networks
SDM: shared decision making
